# Tocilizumab in the Treatment of Chronic Antibody-Mediated Rejection Post Kidney Transplantation: Clinical and Histological Monitoring

**DOI:** 10.3389/fmed.2021.790547

**Published:** 2021-12-24

**Authors:** Johan Noble, Diane Giovannini, Reda Laamech, Farida Imerzoukene, Bénédicte Janbon, Laura Marchesi, Paolo Malvezzi, Thomas Jouve, Lionel Rostaing

**Affiliations:** ^1^Nephrology, Hemodialysis, Apheresis and Kidney Transplantation Department, University Hospital Grenoble, Grenoble, France; ^2^University Grenoble Alpes, Grenoble, France; ^3^Pathology Department, University Hospital Grenoble, Grenoble, France

**Keywords:** kidney transplantation, tocilizumab, chronic antibody-mediated rejection, eGFR, kidney allograft biopsy

## Abstract

**Introduction:** Chronic antibody-mediated rejection (cAMR) has very few effective therapeutic options. Interleukin-6 is an attractive target because it is involved in inflammation and humoral immunity. Therefore, the use of tocilizumab (anti-IL6 receptor, TCZ) is a potential valuable therapeutic option to treat cABMR in kidney-transplant (KT) recipients.

**Materials and Methods:** This single-center retrospective study included all KT recipients that received monthly TCZ infusions in the setting of cABMR, between August 2018 and July 2021. We assessed 12-month renal function and KT histology during follow-up.

**Results:** Forty patients were included. At 12-months, eGFR was not significantly different, 41.6 ± 17 vs. 43 ± 17 mL/min/1.73 m^2^ (*p* = 0.102) in patients with functional graft. Six patients (15%) lost their graft: their condition was clinically more severe at the time of first TCZ infusion. Histological follow-up showed no statistical difference in the scores of glomerulitis, peritubular capillaritis, and interstitial fibrosis/tubular atrophy (IFTA). Chronic glomerulopathy score however, increased significantly over time; conversely arteritis and inflammation in IFTA ares improved in follow-up biopsies.

**Conclusion:** In our study, the addition of TCZ prevented clinical and histological worsening of cABMR in KT recipients, except for more severely ill patients. Randomized studies are needed to clarify the risk/benefit of TCZ in cABMR.

## Introduction

Kidney transplantation remains the best therapeutic option regarding end-stage kidney disease for various reasons including improved survival and quality-of-life compared to those receiving dialysis therapy (1–4) However, the half-life of kidney transplants has not changed that much within the last decades at least in the United States as demonstrated by Lamb *et al*. This was particularly the case in low-risk populations like living-donor-recipients where half-life was 11.4 years in 1989 and 11.9 years in 2005. Finally, within this time-frame first-year attrition rates show dramatic improvements, whereas attrition rates beyond the first year show only small improvements (5). In 2012 Sellarés *et al*. have shown in a prospective cohort of 315 allograft recipients who underwent indication biopsies at 6 days to 32 years posttransplant that many actual failures after indication biopsies manifest phenotypic features of antibody-mediated (ABMR) or mixed rejection. They also underscore the major role of non-adherence (6). Indeed, non-adherence is a major risk factor for developing *de novo* donor-specific alloantibody (dnDSA) after kidney transplantation (7). dnDSA formation at post-transplantation is the leading cause of developing chronic antibody-mediated (cABMR) rejection (8, 9); they have a particularly worse impact upon kidney allograft survival especially when they bind complement, i.e., either C1q (9, 10) or C3d (11) components. Recently, Mayrdorfer *et al*. have reassessed the causes of kidney allograft failure in the modern immunosuppression era in a prospective cohort of 1,642 kidney transplant (KTx) recipients (12). They found that in 51.2% of patients with allograft failure, more than one cause was involved. The most frequent primary or secondary causes leading to graft failure were intercurrent medical events in 36.3% of graft failures followed by T cell-mediated rejection (TCMR) in 34% and ABMR in 30.7%. In 77.9%, a primary cause could be attributed to graft loss, of which ABMR was the most frequent etiology (21.5%).

At present, when cABMR is diagnosed we are left with very few therapeutical options. In a randomized controlled trial, Eskandary *et al*. reported, in cases of donor-specific alloantibody (DSA)-positive chronic ABMR, that bortezomib therapy was no more efficient than a placebo at changing the decline in slope of the estimated glomerular-filtration rate (eGFR) (13). Likewise, Moreso *et al*. reported, in a randomized controlled trial in the setting of cABMR with transplant glomerulopathy (TG) that the combination of polyclonal intravenous immunoglobulins (IVIG) and rituximab, as compared to a placebo, did not significantly modify the natural history of cABMR regarding transplant glomerulopathy (14).

Indeed, in the setting of cABMR, IL-6 is an attractive target. IL-6 is a multifunctional pleiotropic cytokine that stimulates B- and T-cell functions (15, 16). Uncontrolled studies have suggested that blocking IL-6 receptor by tocilizumab (TCZ) might be valuable in patients presenting with cABMR (17) or with transplant glomerulopathy (18). Recently, Doberer *et al*. reported on a phase 2 randomized pilot trial to evaluate the safety (primary endpoint) and efficacy (secondary endpoint) analysis of the anti-IL-6 antibody clazakizumab in 20 KTx patients presenting with late cABMR (19). These data suggested a potentially beneficial effect of anti-IL-6 blockade on cABMR activity and progression.

In the present single-center study, we report on the long-term effects on tocilizumab monthly therapy in kidney transplant recipients presenting with cABMR and/or transplant glomerulopathy.

## Materials and Methods

### Study Population

In this single-center retrospective study, we enrolled all KT recipients that received TCZ in the setting of chronic ABMR, between August 2018 and July 2021. All patients included had to meet the last Banff criteria for chronic active ABMR, category 2 of 2019 Banff classification (20).

All patients received intravenous TCZ at the dose of 8 mg/kg every month scheduled for 6 months. At that point, continuation or discontinuation of TCZ was reassessed every 6 months at the discretion of attending physicians.

We collected demographic data on donor and recipients in the hospital's medical electronic records. The research protocol was approved by the local ethical committee. All medical data were collected from our database [CNIL (French National committee for data protection) approval number 1987785v0].

### Study Design

We assessed in this study the clinical, biological and kidney graft histological data at baseline, month +6 (M6) and month +12 (M12). Baseline was defined at the time of the kidney biopsy that resulted in TCZ introduction for each patient. Usually, follow-up kidney biopsies are performed every 6 months after starting TCZ therapy in order to assess whether TCZ is continued or not.

Data collected were serum creatinine level (μmol/L), eGFR (mL/min/1.73 m^2^), proteinuria or albuminuria (g/L), presence of DSA and their mean fluorescence intensity (MFI), and histological Banff scores: chronic glomerulopathy score (cg), glomerulitis score (g) interstitial fibrosis and tubular atrophy (IFTA) inflammation in areas of interstitial fibrosis and tubular atrophy (i-IFTA), peritubular capillaritis score (ptc), interstitial inflammation (i), tubulitis (t), intimal arteritis (v), staining for C4d on endothelial cells of ptcs and medullary *vasa recta* (c4d), arterial intimal thickening (cv), mesangial matrix expansion (mm), arteriolar hyalinosis (ah), hyaline arteriolar thickening (aah), and total inflammation (ti).

After kidney transplantation anti-HLA antibodies and DSA are assessed on a yearly basis since 2007 and at each time we perform a for-cause kidney biopsy. Anti-HLA antibodies. and DSA are assessed by a Luminex assay using the Immuncor^Ⓡ^ platform. eGFR was calculated using the Chronic Kidney Disease Epidemiology collaboration equation (CKD-EPI).

### Immunosuppression

At the time of kidney transplantation, induction therapy consisted in anti-thymocyte globulin for all patients (Sanofi, Lyon, France). All patients received 1 g of mycophenolate mofetil (MMF) pre-operatively, followed by MMF 2 g/day up to POD 15, then tapered to 1 g/day. Prednisolone was given at the dose of 500 mg pre-operatively, tapered to 10 mg/day at day 30 post-kidney transplantation. If kidney allograft surveillance biopsy at 3 months post-transplant was normal prednisone was stopped. Tacrolimus was started at day 4 post-kidney transplantation and adjusted to achieve trough levels of 8–12 ng/mL the first month, and then 4–8 ng/mL.

### Endpoint

The primary endpoint was to assess the evolution of renal function (eGFR) at M6 and M12 post-TCZ therapy in the setting of cABMR. Patients who have lost their graft within the first year after starting TCZ therapy were excluded from this analysis.

Secondary endpoints were the evolution of the Banff scores (according to the 2019 Banff classification) in kidney allograft biopsies post-TCZ therapy. We performed two analyses. First we merge all follow-up biopsies during the first year to realize paired comparison between baseline and First year biopsies post TCZ. Then, we assessed all biopsies available, whatever the time post-TCZ therapy and grouped them into 4 periods according to quartile time post-TCZ therapy: biopsies done <5 month, between ≥5– <9 months, between ≥9– <17 months and ≥17 months. The first index biopsy, i.e., the one allowing cABMR diagnosis was not included in follow-up analyses. Biopsies of patients that have lost their graft were included in the analyses. Finally, the incidence of allograft failure, i.e., graft loss was also assessed.

### Statistical Analyses

Quantitative data are shown as means ± standard deviations (SD) or as medians with quartiles [Q1–Q3]. Qualitative data are shown as numbers and percentages. We decided to remove missing data for percentages calculation. The chi-squared test was used for categorical variables, especially the comparison of Banff scores between time periods. For the primary endpoint, eGFR values follow a normal distribution. Equality of the variance was also assessed and confirmed. We used a paired student *t*-test to compare eGFR at baseline vs. M6 and M12. For the Banff scores, we first did a global assessment of follow-up scores using a Kruskal-Wallis test. Then, a paired Wilcoxon test was used to compare Banff scores at baseline vs. follow-up, in a matched manner for each patient. A two-sided *p*-value of <0.05 was considered statistically significant. Statistical analyses were conducted using R statistical software.

## Results

### Study Population

Between August 2018 and July 2021, 40 patients received TCZ for cABMR post-kidney transplantation. The baseline characteristics of these patients are summarized in Table 1.

**Table 1:** Demographic characteristics of included KT recipients.

	**Included KT recipients (*n* = 44)**
**Recipient age at KT, years** Mean ± SD	43 ± 15
**Gender (female)**, N (%)	16 (40)
**Living donor**, N (%)	7 (17,5)
**ABO incompatible KT**, N (%)	2 (5)
**HLA incompatible KT**, N (%)	12 (30)
**Main nephropathies**	
- Glomerular disease	11 (27,5)
- Polycystic disease	6 (15)
- Malformative uropathy	6 (15)
**Transplantation rank**	
- First	30 (75)
- Second	9 (22.5)
- Third	1 (2.5)

*KT, Kidney Transplantation; HLA, Human Leukocyte antigen*.

Immunosuppression at the time of cABMR consisted in tacrolimus for 34 patients (85%), MMF for 34 patients (85%), mTOR inhibitors for 6 patients (15.4 %), belatacept for 6 patients (15.4 %) and steroids for 25 patients (62.5%). DSAs were found in 22 patients (55%). Nineteen patients had class II DSAs whereas 7 patients had class I DSAs. For the 18 patients without detectable DSA, cABMR diagnostic was based on histological findings. All those 18 patients had a cg score >0 and a C4d staining in peritubular capillaritis and a [g+cpt] score ≥ 2.

Median time to cABMR diagnostic was 18.9 (6–55) months. TCZ was started after a median time of 34 days (23–155) after cABMR diagnosis. Median follow-up after cABMR diagnosis was 7 (4–13) months.

### Immunosuppression

In some patient the diagnosis of cABMR had been established months/years before starting TCZ therapy. Therefore, some had received in the past either rituximab (*n* = 16; 40%), plasmapheresis (*n* = 8; 20%), high dose of steroids (*n* = 21; 52.5%) or antithymoglobulin (*n* = 2; 5%). For seven patients (17.5%) TCZ therapy was the first line therapy for cABMR.

Immunosuppressive regimen consisted in tacrolimus and mycophenolate acid for all patients at the time of rejection except for one patient that received belatacept and tacrolimus. Post-rejection, 18 patients (45%) were converted to a belatacept-based immunosuppression in association with tacrolimus (4 patients) or mycophenolate mofetil (14 patients). The other patients remained on tacrolimus and mycophenolate mofetil.

### Renal Function

**Figure 1:**
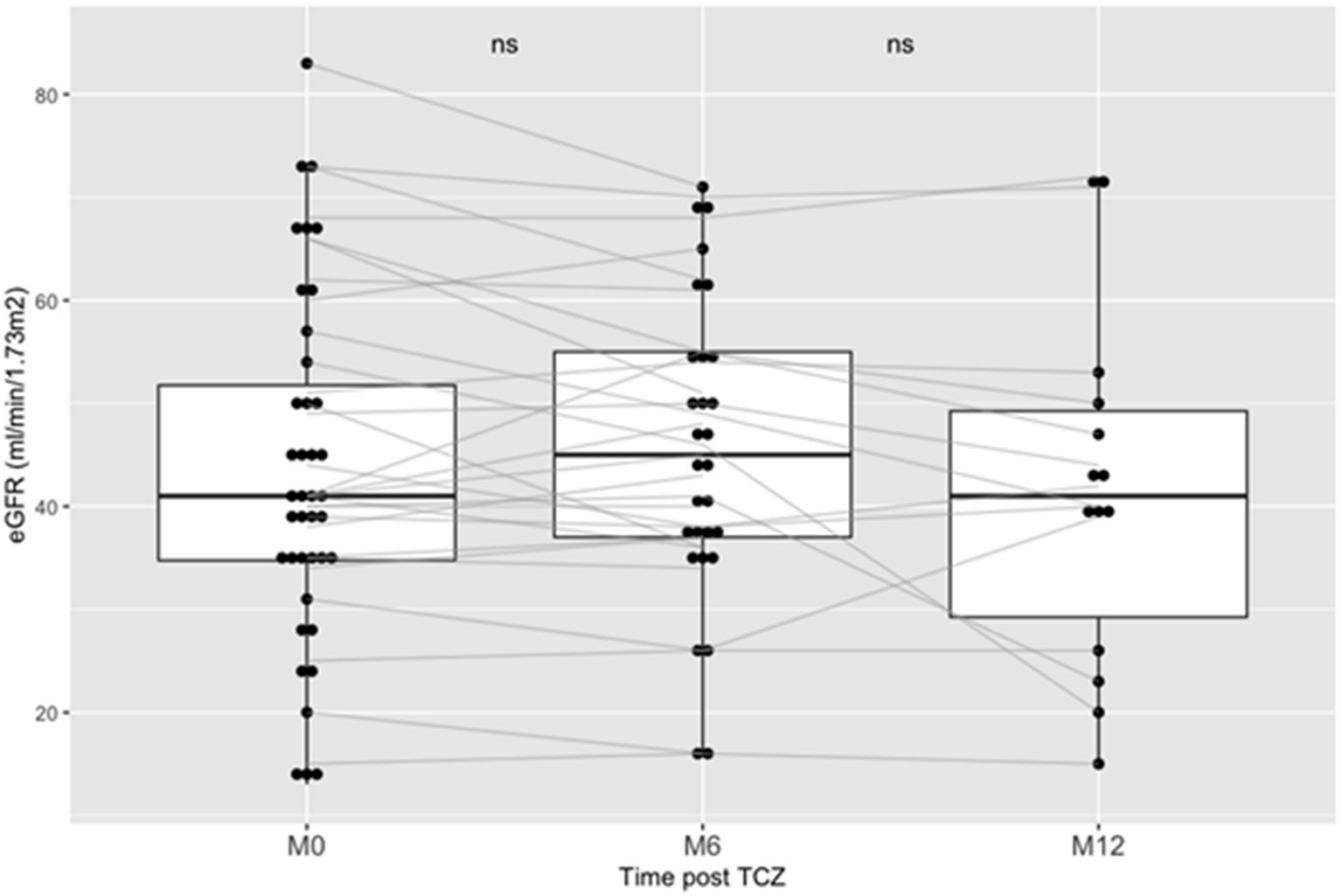
Outcome of eGFR post Tocilizumab in kidney transplanted patients treated for chronic ABMR. Boxplots shows the eGFR (CKD-Epi) of patients at baseline of the introduction of TCZ, at Month-6 (M6) and at Month-12 (M12). TCZ stands for Tocilizumab. AMBR stands for antibody-mediated rejection.

Our primary endpoint was to assess the evolution of eGFR after TCZ treatment. At the time of cABMR diagnosis, mean eGFR was 43 ± 17 mL/min/1.73 m^2^. At M6 post-TCZ therapy, mean eGFR was not significantly different, i.e., 45.3 ± 15 mL/min/1.73 m^2^ (*p* = 0.12). Similarly, at M12 post-TCZ therapy, mean eGFR was not significantly different as compared to baseline, i.e., 41.6 ± 17 mL/min/1.73m^2^ (*p* = 0.102). Figure 1 shows the evolution of eGFR between baseline and M12 for each patient. We then assessed the eGFR slopes between M0 and M6 and between M0 and M12 using a linear model regression: we did not find a significant difference in eGFR (*p* = 0.56 between M0-M6 and *p* = 0.77 between M0-M12).

However, when we included in the analyses an eGFR = 5 ml/min/1.73 m^2^ for the 5 patients who lost their graft within the 12 months post-TCZ, eGFR was 40 ± 19 ml/min.1.73m^2^ at M6 post-TCZ (*p* = 0.01 as compared to baseline) and eGFR was 32 ± 21 ml/min.1.73m^2^ at M12 post-TCZ (*p* = 0.02 as compared to baseline).

Regarding proteinuria, mean proteinuria at baseline was 1.0 ± 0.9 g/L. At M6 and M12 post-TCZ therapy mean proteinuria were 0.8 ± 1.1 g/l. and 0.9 ± 1.1 g/L, statistically not different from baseline, respectively *p* = 0.95 and *p* = 0.28.

Six patients (15%) lost their graft after an average time of 2.5 [2.5 – 6.5] months post-cABMR diagnostic. These patients had a significantly lower eGFR at the time of cABMR diagnostic (first biopsy) as compared to other patients: 24.5 ± 16 vs. 46.3 ± 15 ml/min/1.73 m^2^ (*p* = 0.006). Similarly, their proteinuria level was significantly higher at the time of cABMR diagnostic, i.e., 1.8 ± 1 vs. 0.8 ± 0.9 g/L (*p* = 0.022). Finally, they had at baseline a more severe histological presentation, i.e., with ct = 3, ci = 3 and v = 2 than in those who did not lose their graft (*p* = 0.007, *p* = 0.002 and *p* = 0.001 respectively).

### Histologic Evolution

From the 44 patients, 38 (70%) have had a follow-up kidney biopsy after TCZ introduction and 28 (63.6%) had at least 2 kidney biopsies post-TCZ. Average times of kidney biopsies were: 3.1 (1–5) months, 4.2 [1.2 – 10] months, 4.2 [1.9 – 6.7] months, 6.8 [3.5 – 9.4] months for the second, third, fourth and fifth biopsy post-TCZ respectively. Median time between follow-up biopsies was 173.5 (105–224) days.

At baseline, half of biopsies had a glomerulitis score (g) ≥2 and a peritubular capillaritis score (ptc) ≥2. Twenty five percent had a chronic glomerulopathy score (cg) ≥ 2. Eighteen percent of patients had an IFTA score ≥2 at baseline.

We assessed the significant modification of Banff score between biopsies post-TCZ therapy.

First, we compared the Banff scores between baseline and first-year follow-up post-TCZ treatment in 20 paired patients (Table 2). There were no statistical differences in the acute and chronic histologic scores of those patients in the follow-up biopsies as compared to diagnostic biopsies.

**Table 2:** Histological Banff scores during the first year post-TCZ treatment for cAMR.

**Banff Scores**	**Baseline (*N* = 20)**	**5–12 months (*N* = 20)**	*p* value^*^
**Acute scores:** ***N*** (%)^**^
**t score**			0.595
0	12 (60.0%)	13 (65.0%)	
1	7 (35.0%)	7 (35.0%)	
2	1 (5.0%)	0 (0.0%)	
**i score**			0.220
0	18 (90.0%)	19 (95.0%)	
1	0 (0.0%)	1 (5.0%)	
2	2 (10.0%)	0 (0.0%)	
**v scores**			0.311
0	18 (94.7%)	19 (100.0%)	
1	1 (5.3%)	0 (0.0%)	
N-Miss	1	1	
**g scores**			0.968
0	4 (20.0%)	4 (21.1%)	
1	6 (30.0%)	5 (26.3%)	
2	10 (50.0%)	10 (52.6%)	
N-Miss	0	1	
**ptc scores**			0.361
0	3 (15.0%)	6 (30.0%)	
1	3 (15.0%)	1 (5.0%)	
2	14 (70.0%)	13 (65.0%)	
**Chronic scores:** ***N*** (%)^**^
**cg**			0.683
0	16 (80.0%)	13 (68.4%)	
1	1 (5.0%)	2 (10.5%)	
2	3 (15.0%)	4 (21.1%)	
N-Miss	0	1	
**cv**			0.833
0	7 (36.8%)	5 (27.8%)	
1	4 (21.1%)	4 (22.2%)	
2	8 (42.1%)	9 (50.0%)	
N-Miss	1	2	
**IFTA**			0.512
0	5 (31.2%)	9 (47.4%)	
1	7 (43.8%)	5 (26.3%)	
2	4 (25.0%)	5 (26.3%)	
N-Miss	4	1	
**iIFTA**			0.161
0	5 (25.0%)	10 (52.6%)	
1	6 (30.0%)	5 (26.3%)	
2	9 (45.0%)	4 (21.1%)	
N-Miss	0	1	

* *p-value compares patient paired Banff scores of biopsies between baseline and 5–12 months post-TCZ therapy*.

** *Missing values are removed from the percentage calculation*.

The Table 3 shows the global Banff scores of all patients according to biopsies performed during quartile times post-TCZ therapy: <5 month, ≥5– <9 months, ≥9– <17 months and ≥17 months. We then compared the Banff scores between baseline and all period of follow-up post-TCZ treatment in all patients. There was no statistical difference in the follow-up histologic scores except for the intimal arteritis score (v) = 1 that was present in 36 % in biopsies performed <5 months but was not found in later biopsies (*p* = 0.001). Also, the number of biopsies with a cg score ≥2 significantly increased over time, i.e., 0% in biopsies <5 months and 50% in biopsies >12 months (*p* = 0.037). There was no statistical difference during the follow-up biopsies for the IFTA, i-IFTA, g, ptc, i, t, and cv scores suggesting a relative stability of the histological lesions.

**Table 3:** Histological Banff scores comparison post TCZ treatment for cAMR.

**Banff scores**	**Baseline *N* = 40**	** <5 months *N* = 12**	**5–9 months *N* = 14**	**9–17 months *N* = 14**	**≥17 months *N* = 16**
**Acute scores:** ***N*** (%)^**^					
**i score**					
0	36 (92)	9 (75)	12 (92)	14(100)	14 (87)
1	1 (3)	2 (17)	1 (8)	0	1 (6)
≥2	2 (5)	1 (8)	0	0	1 (6)
**t score**					
0	26 (65)	5 (42)	7 (54)	11 (79)	9 (56)
1	11 (27)	5 (42)	6 (46)	2 (14)	6 (37)
≥2	3 (7)	2 (17)	0	1 (7)	1 (6)
**v score**					
0	34 (92)	7 (64)	12 (100)	14(100)	14 (100)
1	3(8)	4 (36)	0	0	0
**g score**					
0	8 (20)	3 (27)	4 (33)	2 (15)	1 (8)
1	10 (25)	3 (27)	2 (17)	3 (23)	1 (8)
≥2	22 (55)	5 (45)	6 (50)	8 (61)	11 (85)
**ptc score**					
0	11 (27)	4 (33)	4 (31)	5 (36)	2 (12)
1	7 (17)	3 (25)	1 (8)	2 (14)	5 (31)
≥2	22 (55)	5 (42)	8 (61)	7 (50)	9 (56)
Chronic scores: N (%)^**^					
**IFTA score**					
0	11 (33)	4 (40)	6 (50)	6 (43)	2 (13)
1	16 (48)	4 (40)	2 (17)	7 (50)	9 (60)
≥2	6 (18)	2 (20)	4 (33)	1 (7)	4 (27)
**i-IFTA score**					
0	11 (29)	2 (18)	6 (46)	6 (46)	4 (25)
1	12 (32)	7 (64)	3 (23)	6 (46)	8 (50)
≥2	15 (39)	2 (18)	4 (31)	1 (8)	4 (25)
**cg score**					
0	27 (67)	10 (91)	8 (61)	9 (75)	5 (31)
1	3 (7)	1 (9)	2 (15)	2 (17)	3 (19)
≥2	10 (25)	0	1 (8)	1 (8)	8 (50)
**cv score**					
0	12 (33)	4 (36)	4 (33)	2 (15)	0
1	6 (17)	4 (36)	3 (25)	4 (31)	4 (27)
≥2	8 (50)	3 (27)	5 (42)	7 (54)	11 (73)

** *Missing values are removed from the percentage calculation*.

Evolution of biopsy scores for each patient are shown using alleviate figures for IFTA (Supplementary Figure 1), for cg (Supplementary Figure 2) for g (Supplementary Figure 3) and ptc scores (Supplementary Figure 4). We assessed more precisely the outcome by using a paired Wilcoxon test to compare the evolution of Banff scores between baseline and each period of follow-up for each patient:

For the cg score, the average cg score was higher in >17 months biopsies as compared to baseline, i.e., 2.4 ± 0.8 vs. 1.5 ± 0.9, but not statistically significant (*p* = 0.053). The arterial intimal thickening (cv) score was also higher in follow-up biopsies as compared to baseline, i.e., 1.7 ± 0.4 vs. 1.2 ± 0.9 respectively, also without reaching significance (*p* = 0.056).

However, the iIFTA score seemed to decrease with time, i.e., in 9–12 months biopsies, iIFTA score was 0.6 ± 0.6 vs. 1.1 ± 0.8 at baseline (*p* = 0.053).

There was no statistical difference between baseline and follow-up biopsies (<5 months, 5–9 months, 9–17 months and >17 months) for the IFTA, the glomerulitis (g) the peritubular capillaritis (ptc), the interstitial inflammation (i), the tubulitis (t) and the intimal arteritis (v) scores (data not shown).

Then, we assessed the evolution of patients with an history of circulating DSA as compared to DSA(−) patients. We compared g, cg, IFTA, iIFTA, and ptc scores in each follow-up biopsy post-TCZ therapy (second biopsy, third biopsy, fourth biopsy, fifth biopsy and sixth biopsy post-TCZ). There were no statistical differences in the Banff score between DSA(+) and DSA(−) patients except for the ptc score after the third biopsy in which a score ≥2 was present in 6 patients (66%) in DSA(+) group as compared to 1 patient (16%) in the DSA(−) group (*p* = 0.03). Four DSA (−) patients have lost their graft at the end of follow-up (22%). In the DSA(+) group, 2 patients (9%) have lost their graft. The difference did not reach significance (*p* = 0.476).

## Discussion

In this study, we assessed the clinical and histological outcomes of kidney transplant recipients experiencing chronic active ABMR treated with IV tocilizumab. We have shown that at 6 months post-TCZ and 12 months post-TCZ, renal function and proteinuria remained stable in patients with a functional graft. However, eGFR was significantly lower at 12 months if we included the value of 5 ml/min/1.73 m^2^ for patients who have lost their kidney graft within the first year. We have also assessed the evolution of Banff scores in follow-up kidney allograft biopsies and showed that the parameters of microvascular inflammation (g and ptc) and the chronicity markers (cg, IFTA, i-IFTA) also remained stable during the follow-up. To summarize, although a control group is missing, we assume that TCZ therapy may have allowed preventing clinical and histological worsenings in the setting of cABMR in our cohort.

IL-6 has pleiotropic effects upon the inflammatory response. Sites of action are as numerous as the possible therapeutic fields of tocilizumab: impact on hematopoiesis and on keratocyte proliferation, differentiation of osteoclasts (21). Regarding the humoral response, IL-6 plays a role in the differentiation of the mature B-cell into a cell capable of secreting antibodies (22). It has been showed that TCZ significantly and non-specifically reduce the IgG synthesis in highly sensitized kidney transplant recipients treated for cABMR (23). However, in highly sensitized kidney transplant candidates, tocilizumab as a monotherapy limited B cell maturation but however, it had almost no effect on anti-HLA alloantibodies (24). Chandran et al. conducted a randomized controlled clinical trial of clinically stable kidney transplant recipients on calcineurin inhibitor, mycophenolate mofetil, and prednisone, with subclinical graft inflammation noted on surveillance biopsies during the first-year post-transplant: they were randomized to receive either TCZ (8 mg/kg every 4 weeks, six infusions) or placebo. They showed an increase of circulating Treg as compared to controls, and tocilizumab-treated subjects were more likely to show improved Banff ti-score (62.5% vs. 21.4%, *p* = 0.03) (25).

Two recent studies addressed the use of IV tocilizumab for treating cABMR. Lavacca et al. included fifteen cABMR patients for which the first-line therapy was tocilizumab (18). They were followed for a median time of 20.7 months. They found that despite the majority of patients experienced advanced transplant glomerulopathy (TG) at diagnosis (60% with cg3), glomerular filtration rate and proteinuria stabilized during the follow-up, with a significant reduction in donor-specific antibodies. In addition, protocol biopsies after 6 months demonstrated significant amelioration of microvascular inflammation and no TG, C4d deposition, or IF/TA progression. Finally, gene-expression and immunofluorescence analysis showed upregulation of three genes (TJP-1, AKR1C3, and CASK) involved in podocyte, mesangial, and tubular restoration. The second single-center retrospective study reported on nine ABMR kidney transplant patients resistant to apheresis, rituximab, and intravenous immunoglobulins that were treated with monthly IV tocilizumab (26). They were compared with a historical cohort of 37 patients with similar clinical, immunological, and histological characteristics. They found that 1-year graft survival and the decline in renal function did not differ between patients who received tocilizumab and those who did not. In addition, histological follow-up showed that despite a decrease in inflammation and tubulitis scores after tocilizumab, the course of antibody-mediated lesions and chronic glomerulopathy were similar in both groups (26).

In our study, the addition of TCZ may have prevented clinical and histological worsening of cABMR in kidney transplant recipients, except for more severely ill patients. Indeed, 6 patients lost their graft. When we assessed the clinical characteristics of these patients at baseline, we showed that they had a significant more severe presentation with a lower eGFR (24 ml/min/1.73 m^2^), higher level of proteinuria (1.8 g/L) and worse histological presentation (ci, ct and v scores). We assume that for these patients TCZ treatment was initiated too late.

Most of our patients had follow-up kidney biopsies within the first-year post-TCZ treatment. We did not observe significant worsening in the Banff scores over time. However, during TCZ therapy most of our patients (34/40) were clinically stable without rejection. Although not reaching the level of statistical significance we observed an improvement of some histological parameters such as peritubular capillaritis.

Recently, Sethi et al. assessed infections occurring among 148 kidney recipients treated with tocilizumab 8 mg/kg IV monthly (*n* = 83) or IVIG/rituximab (*n* = 65) for donor-specific antibodies and antibody-mediated rejection through 1 year after treatment cessation (27). There were 106 infections observed over 190.1 person-years, yielding an incidence rate of 558 infections/1,000 patient-years; however a lower incidence rate of infections was observed among tocilizumab-treated compared with IVIG/rituximab-treated patients (463 infections/1,000 patient-years vs. 730 infections/1,000 patient-years; *P* = 0.02). Twenty-five of 49 infections (51%) in the IVIG/rituximab group required hospitalization compared with 31 of 57 (54%; *P* = 0.85) in the tocilizumab group. Finally, there were no infection-related deaths in either group. On multivariable Poisson regression, there was a lower incidence rate of infections associated with tocilizumab compared with IVIG/rituximab. These findings are very reassuring at using in the long run tocilizumab therapy in kidney transplant recipients.

Our study has several limitations inherent to the retrospective and uncontrolled design. The number of patients in the present study is limited, although no other published study on the subject has included more patients. Large randomized clinical trials are needed to clarify the benefit of TCZ for treating cABMR in solid-organ transplant patients because the safety profile of TCZ therapy in kidney transplant recipients is reassuring (27).

Recent data highlight the potential role of TCZ in controlling the humoral and inflammatory response and its potential benefit in cABMR treatment. The available studies and our study seem to show that it is possible to stabilize the decline in renal function and histological rejection lesions. Indeed, randomized studies are needed in this area, as cABMR suffers from the lack of effective treatments. Blocking the IL-6 pathway by either anti-IL-6 receptor or by anti-IL-6 monoclonal antibodies seems to a relevant avenue.

## Data Availability Statement

The raw data supporting the conclusions of this article will be made available by the authors, without undue reservation.

## Ethics Statement

The study was conducted according to the guidelines of the Declaration of Helsinki and approved by the Ethics Committee of CNIL (French National Committee for Data Protection) approval number 1987785v0. N° BRIF: BB-0033-00069. Informed consent was obtained from all subjects involved in the study.

## Author Contributions

JN, PM, TJ, and LR designed the study. JN, PM, BJ, and LR recruited the patients. DG reviewed the kidney biopsies. RL, FI, LM, and JN collected the data. JN and LR wrote the manuscript. PM and TJ edited the manuscript. All authors contributed to the article and approved the submitted version.

## Conflict of Interest

The authors declare that the research was conducted in the absence of any commercial or financial relationships that could be construed as a potential conflict of interest.

## Publisher's Note

All claims expressed in this article are solely those of the authors and do not necessarily represent those of their affiliated organizations, or those of the publisher, the editors and the reviewers. Any product that may be evaluated in this article, or claim that may be made by its manufacturer, is not guaranteed or endorsed by the publisher.

## Abbreviations

ah: arteriolar hyalinosis
aah: hyaline arteriolar thickening
cABMR: chronic antibody-mediated rejection
cg: chronic glomerulopathy score
cv: arterial intimal thickening
dnDSA: *de novo* Donor Specific Antibody
eGFR: estimated Glomerular Filtration Rate
ESKD: End-stage kidney disease
g: glomerulitis score
i: interstitial inflammation
IFTA: interstitial fibrosis and tubular atrophy
i-IFTA: inflammation in areas of interstitial fibrosis and tubular atrophy
KT: Kidney Transplantation
MFI: mean fluorescence intensity
mm: mesangial matrix expansion
ptc: peritubular capillaritis score
TCMR: T cell-mediated rejection
TCZ: Tocilizumab
t: tubulitis
v: intimal arteritis.
