# Supplementary figures and images for: Tocilizumab in the Treatment of Chronic Antibody-Mediated Rejection Post Kidney Transplantation: Clinical and Histological Monitoring
Source: Front Med (Lausanne). 2021 Dec 24;8:790547. doi: 10.3389/fmed.2021.790547 (PMC8739887; doi:10.3389/fmed.2021.790547)

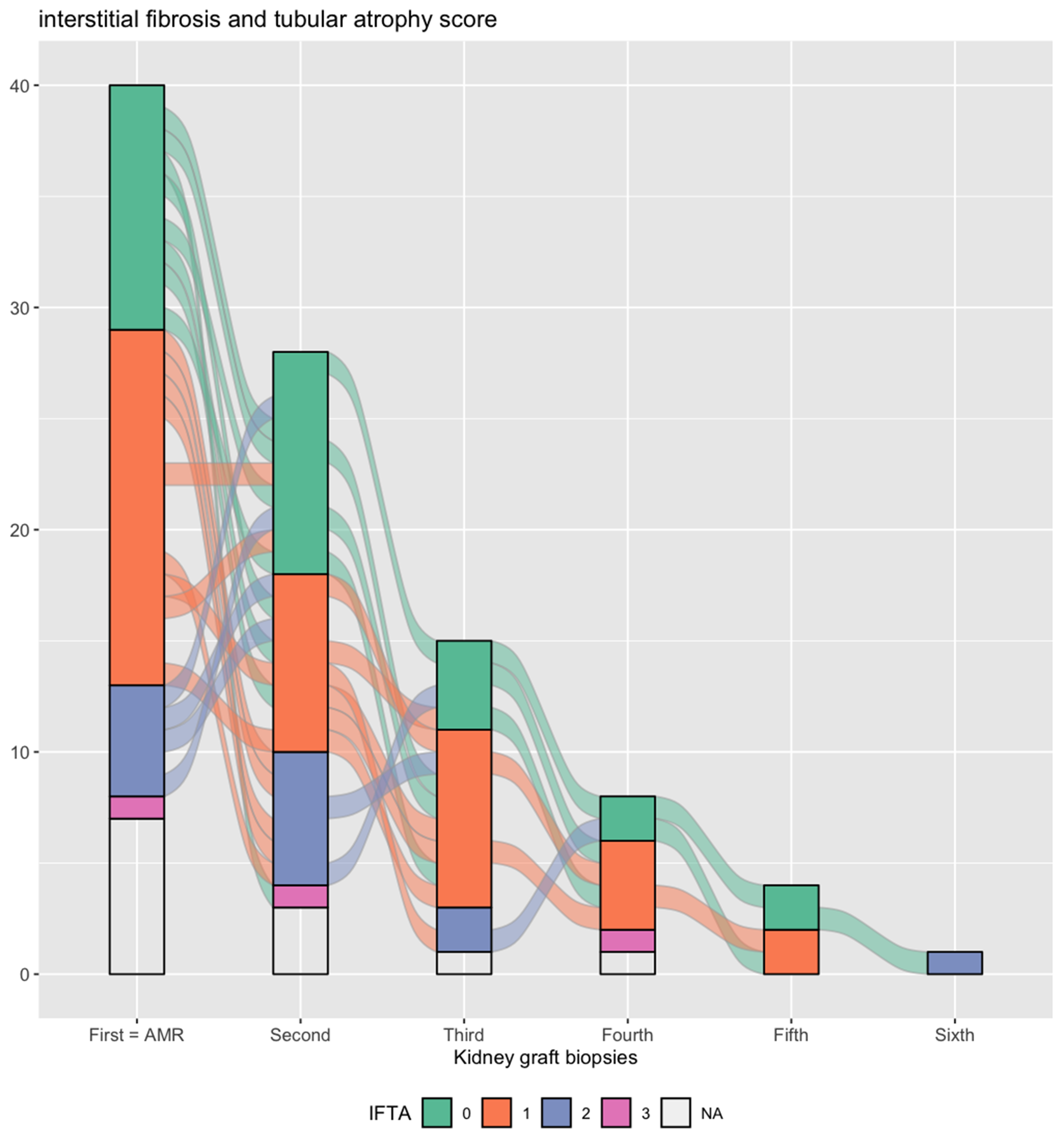

Supplement: Supplementary file 1 [file Image_1.tif]

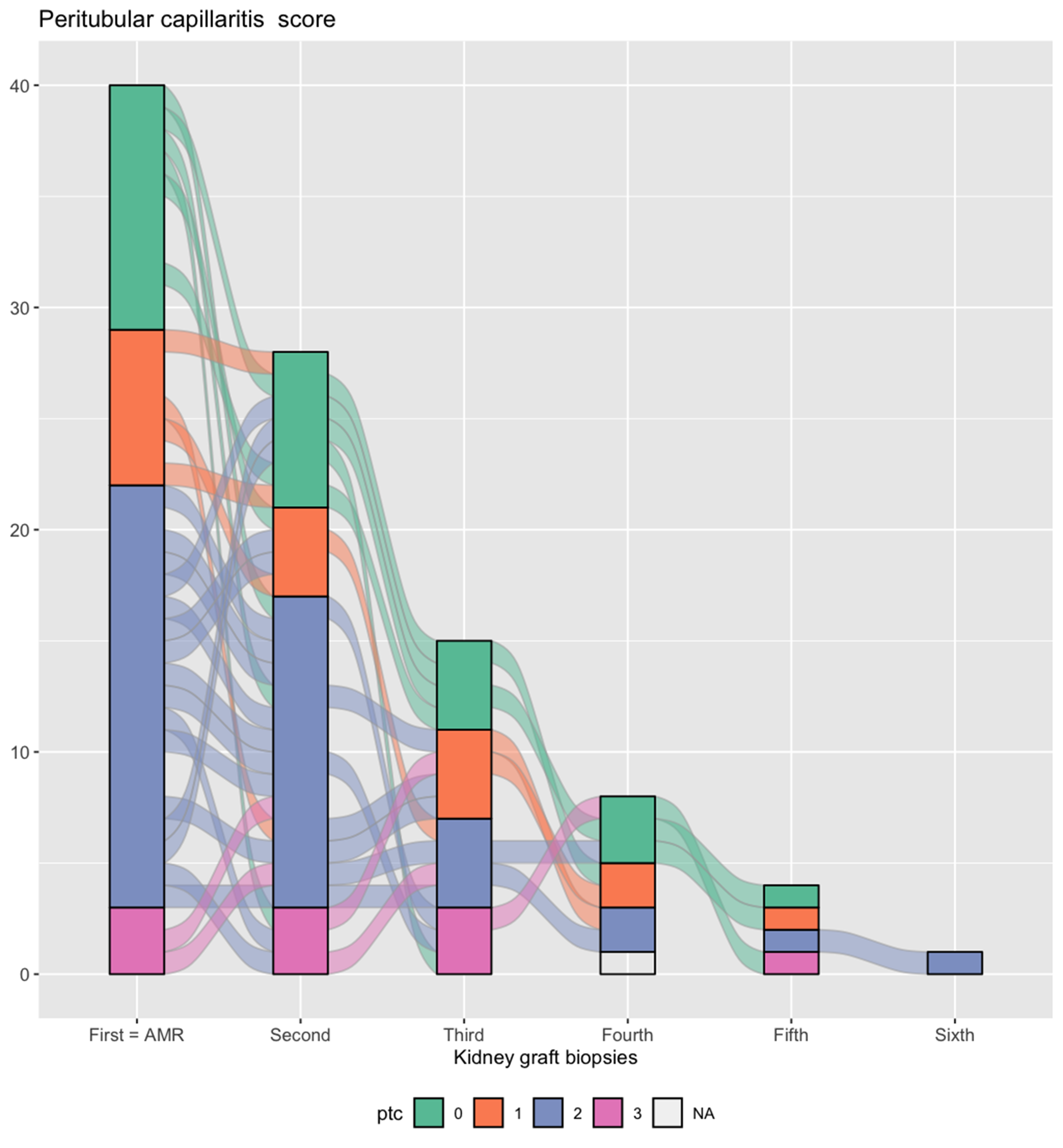

Supplement: Supplementary file 2 [file Image_2.tif]

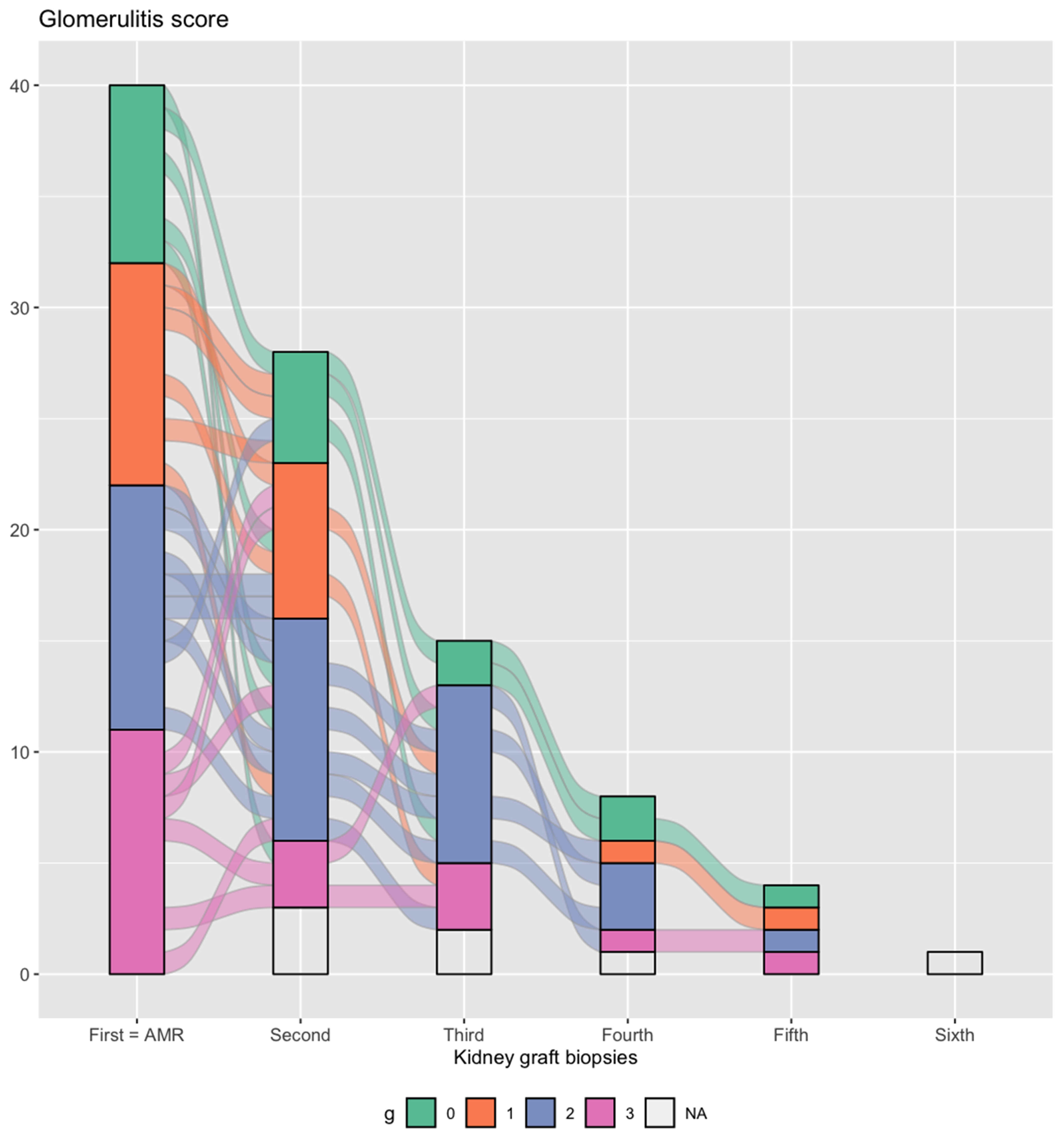

Supplement: Supplementary file 3 [file Image_3.tif]

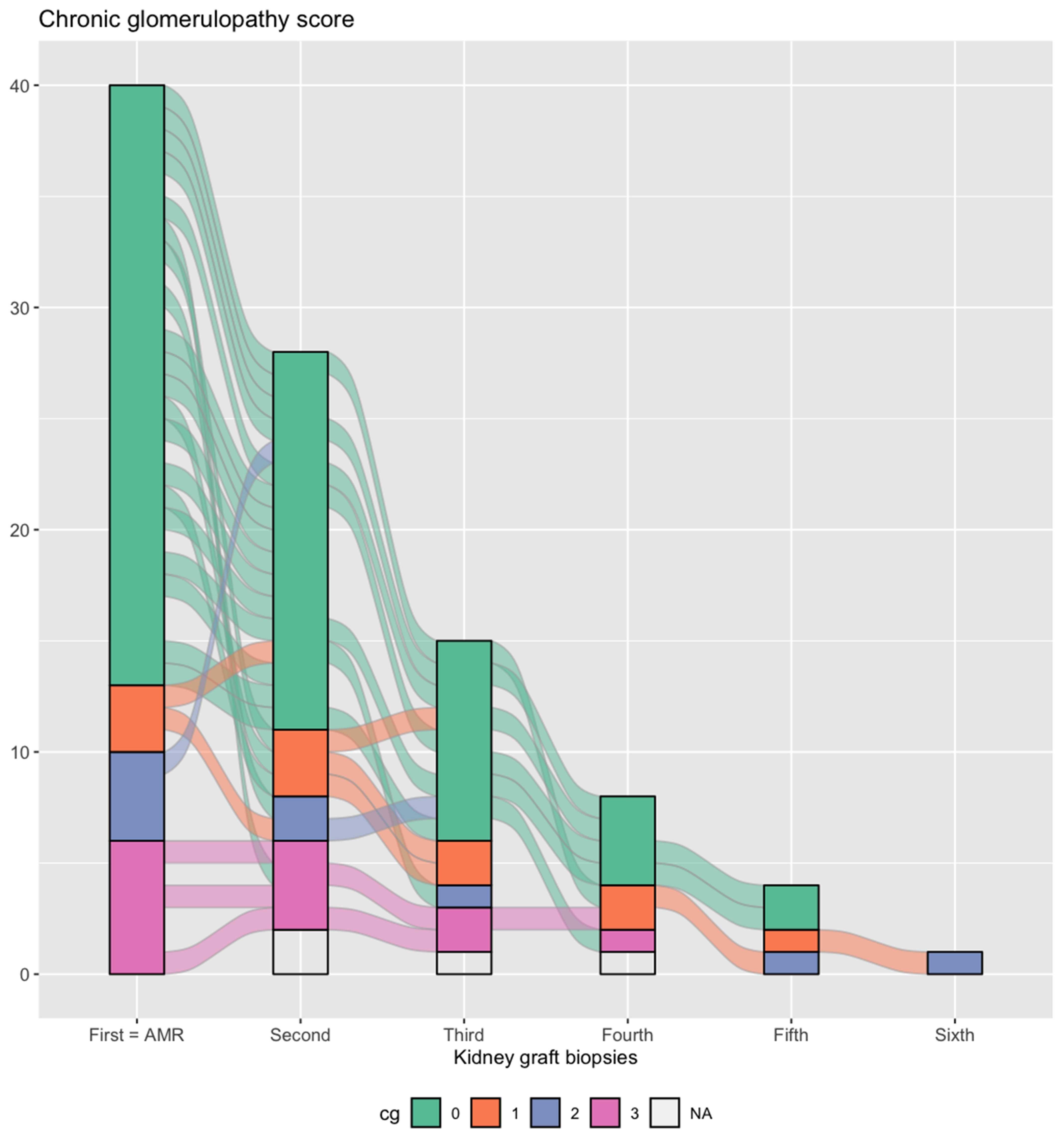

Supplement: Supplementary file 4 [file Image_4.tif]
